# Supplementary material for: Comparative genomics provides new insights into the diversity, physiology, and sexuality of the only industrially exploited tremellomycete: Phaffia rhodozyma
Source: BMC Genomics. 2016 Nov 9;17:901. doi: 10.1186/s12864-016-3244-7 (PMC5103461; doi:10.1186/s12864-016-3244-7)
Supplement: Additional file 6: — List of orphan genes with links to PFAM (related to Additional file 1: Table S1). (ZIP 1428 kb) [file 12864_2016_3244_MOESM6_ESM.zip › BLAST_HTML_FTR/G04795_P.html]

BLAST Search Results


```
BLASTP 2.2.27+


Reference:
Stephen F. Altschul, Thomas L. Madden, Alejandro A. Schäffer,
Jinghui Zhang, Zheng Zhang, Webb Miller, and David J. Lipman (1997),
"Gapped BLAST and PSI-BLAST: a new generation of protein database
search programs", Nucleic Acids Res. 25:3389-3402.


Reference for
composition-based statistics:
Alejandro A. Schäffer, L. Aravind, Thomas L. Madden, Sergei
Shavirin, John L. Spouge, Yuri I. Wolf, Eugene V. Koonin, and
Stephen F. Altschul (2001), "Improving the accuracy of PSI-BLAST
protein database searches with composition-based statistics and
other refinements", Nucleic Acids Res. 29:2994-3005.


Database: nr
           71,551,133 sequences; 26,053,659,533 total letters


Query= G04795_P

Length=445
                                                                      Score     E
Sequences producing significant alignments:                          (Bits)  Value

emb|CDZ96353.1|  hypothetical protein [Xanthophyllomyces dendrorh...   850    0.0  
gb|KIY51758.1|  hypothetical protein FISHEDRAFT_56434 [Fistulina ...  43.5    0.54 


 >emb|CDZ96353.1| hypothetical protein [Xanthophyllomyces dendrorhous]
Length=425

 Score =  850 bits (2195),  Expect = 0.0, Method: Compositional matrix adjust.
 Identities = 423/425 (99%), Positives = 424/425 (99%), Gaps = 0/425 (0%)

Query  20   MTTSIEPSQKALKKPRRSRKKIPPIRPDLPHHEKPRPLKDKSPIPSRPSSLQERISRLIL  79
            MTTSIEPSQKALKKPRRSRKKIPPIRPDLPHHEKPRPLKDKSPIPSRPSS QERISRLIL
Sbjct  1    MTTSIEPSQKALKKPRRSRKKIPPIRPDLPHHEKPRPLKDKSPIPSRPSSSQERISRLIL  60

Query  80   DQRLRFDKLKRRDDMLLIEKICDAPLSQREFIDRHMSKSTKPLSTTHSRRRALLPPIEYF  139
            DQRLRFDKLKRRDDMLLIEKIC+APLSQREFIDRHMSKSTKPLSTTHSRRRALLPPIEYF
Sbjct  61   DQRLRFDKLKRRDDMLLIEKICNAPLSQREFIDRHMSKSTKPLSTTHSRRRALLPPIEYF  120

Query  140  AIWLQTNLVVLIQNIVQRALDDLSVCASRPAYDMVQYPQLFGSPPSLSSSSPARQVSPSI  199
            AIWLQTNLVVLIQNIVQRALDDLSVCASRPAYDMVQYPQLFGSPPSLSSSSPARQVSPSI
Sbjct  121  AIWLQTNLVVLIQNIVQRALDDLSVCASRPAYDMVQYPQLFGSPPSLSSSSPARQVSPSI  180

Query  200  KRLNKKPCETLHDIQLRNLEGILQRVLRLANENRLATQPRIILLHHLQSKIPPTTQVSTE  259
            KRLNKKPCETLHDIQLRNLEGILQRVLRLANENRLATQPRIILLHHLQSKIPPTTQVSTE
Sbjct  181  KRLNKKPCETLHDIQLRNLEGILQRVLRLANENRLATQPRIILLHHLQSKIPPTTQVSTE  240

Query  260  TASYSFLPPETLSKPPCSLSSRPSRTQRSLAKSSPRPIDALWSRYLCMIPYPASPSYKVI  319
            TASYSFLPPETLSKPPCSLSSRPSRTQRSLAKSSPRPIDALWSRYLCMIPYPASPSYKVI
Sbjct  241  TASYSFLPPETLSKPPCSLSSRPSRTQRSLAKSSPRPIDALWSRYLCMIPYPASPSYKVI  300

Query  320  FDSLVWDRDCILLLVDDVQLPKNLPKDPNNKTFDRALWNLPPEGIHDLLSLTVWKSKRVD  379
            FDSLVWDRDCILLLVDDVQLPKNLPKDPNNKTFDRALWNLPPEGIHDLLSLTVWKSKRVD
Sbjct  301  FDSLVWDRDCILLLVDDVQLPKNLPKDPNNKTFDRALWNLPPEGIHDLLSLTVWKSKRVD  360

Query  380  EAHVVNLALKARLDERRAREDFSLKHGDAGVRRPRPIMGVGEVNWLRIGKEQRDITGTFA  439
            EAHVVNLALKARLDERRAREDFSLKHGDAGVRRPRPIMGVGEVNWLRIGKEQRDITGTFA
Sbjct  361  EAHVVNLALKARLDERRAREDFSLKHGDAGVRRPRPIMGVGEVNWLRIGKEQRDITGTFA  420

Query  440  GKSIE  444
            GKSIE
Sbjct  421  GKSIE  425


>gb|KIY51758.1| hypothetical protein FISHEDRAFT_56434 [Fistulina hepatica ATCC 
64428]
Length=1158

 Score = 43.5 bits (101),  Expect = 0.54, Method: Compositional matrix adjust.
 Identities = 31/90 (34%), Positives = 48/90 (53%), Gaps = 7/90 (8%)

Query  30   ALKKPRRSRKKIPPIRPD-LPHHEKPRPLKDKSPIPSRPSSLQERISRLILD---QRLRF  85
            AL+ P+RS   +PP+  +   H  KP  +  K   PS+PSS    + R + D   QRLR 
Sbjct  44   ALRSPKRSASGLPPLTSNPTTHTSKPMTVNRKPMTPSKPSSASIGVPRPLADPHHQRLRE  103

Query  86   DKLKRRDDMLLIEKICDAPLSQREFIDRHM  115
            D L+R   +  ++ +C   LS++E +D  M
Sbjct  104  DALRRVGLLPAVDPMC---LSEQEAVDDRM  130


Lambda      K        H        a         alpha
   0.321    0.135    0.406    0.792     4.96 

Gapped
Lambda      K        H        a         alpha    sigma
   0.267   0.0410    0.140     1.90     42.6     43.6 

Effective search space used: 4375122449841


  Database: nr
    Posted date:  Sep 23, 2015 12:05 AM
  Number of letters in database: 26,053,659,533
  Number of sequences in database:  71,551,133


Matrix: BLOSUM62
Gap Penalties: Existence: 11, Extension: 1
Neighboring words threshold: 11
Window for multiple hits: 40
```
